# The Clinical Course of Acute Pancreatitis and the Inflammatory Mediators That Drive It

**DOI:** 10.1155/2012/360685

**Published:** 2012-12-12

**Authors:** Leena Kylänpää, Zoltán Rakonczay, Derek A. O'Reilly

**Affiliations:** ^1^Department of Surgery, Helsinki University Central Hospital, Haartmaninkatu 4, 00290 Helsinki, Finland; ^2^First Department of Medicine, University of Szeged, P.O. Box 427, Szeged 6701, Hungary; ^3^Department of Surgery, North Manchester General Hospital, Delaunays Road, Manchester M8 5RB, UK; ^4^Cancer Studies Research Group, The University of Manchester, Manchester Academic Health Science Centre, Manchester, UK

## Abstract

Acute pancreatitis (AP) is a common emergency condition. In the majority of cases, it presents in a mild and self-limited form. However, about 20% of patients develop severe disease with local pancreatic complications (including necrosis, abscess, or pseudocysts), systemic organ dysfunction, or both. A modern classification of AP severity has recently been proposed based on the factors that are causally associated with severity of AP. These factors are both local (peripancreatic necrosis) and systemic (organ failure). In AP, inflammation is initiated by intracellular activation of pancreatic proenzymes and/or nuclear factor-**κ**B. Activated leukocytes infiltrate into and around the pancreas and play a central role in determining AP severity. Inflammatory reaction is first local, but may amplify leading to systemic overwhelming production of inflammatory mediators and early organ failure. Concomitantly, anti-inflammatory cytokines and specific cytokine inhibitors are produced. This anti-inflammatory reaction may overcompensate and inhibit the immune response, rendering the host at risk for systemic infection. Currently, there is no specific treatment for AP. However, there are several early supportive treatments and interventions which are beneficial. Also, increasing the understanding of the pathogenesis of systemic inflammation and the development of organ dysfunction may provide us with future treatment modalities.

## 1. Introduction

Acute pancreatitis (AP) is a disease of varied etiology, yet each produces a similar pattern of disease, indicating that they all converge at a common point, to initiate a cascade of events resulting in AP [1, 2]. The overwhelming evidence indicates that this common event involves the premature activation and retention of proteases within the acini which causes cellular injury [3]. In parallel or alternatively to these events, the proinflammatory transcription factor nuclear factor-*κ*B (NF-*κ*B) is activated resulting in the upregulation in expression of cytokines and chemokines [4]. Consequently, recruitment of inflammatory cells, such as neutrophils and macrophages, takes place [5]. This further amplifies the inflammatory reaction and the extent of pancreatic injury. The degree to which these mediators escape into the circulation determines the nature of the systemic inflammatory response. Finally, if the resolution fails to occur, pancreatic infection may supervene.

This paper outlines the clinical course of AP, especially the systemic inflammation and the key mediators that underpin it. We detail the importance of organ failure to outcome. Finally, we speculate upon the future prospects for immunomodulating treatments to act as therapeutic damage-control agents.

## 2. The Clinical Course of Acute Pancreatitis

According to the Atlanta classification, severe AP is defined by the presence of local complications and/or organ failure (shock, pulmonary insufficiency, and renal failure) [6]. The Atlanta classification has been criticized because it failed to discriminate between patient subgroups with different outcomes; for example, it categorized patients with a local complication and a favorable outcome as severe [7]. Hence, a determinant-based classification of AP severity has recently been proposed [8]. This classification is principally based on the factors that are causally associated with severity of AP. These factors are called “determinants” and they are both local and systemic. The local determinant of severity is necrosis of the pancreas and/or peripancreatic tissue termed *(peri)pancreatic necrosis.* The systemic determinant of severity is covered by the term *organ failure. Organ failure* is defined for 3 organ systems (cardiovascular, renal, and respiratory) using the SOFA (Sepsis-Related Organ Failure Assessment) score [9] or when the relevant threshold is breached, as follows:

- *cardiovascular*: need for inotropic agent,
- *renal*: creatinine ≥ 171 **μ**mol/L (≥ 2.0 mg/dL),
- *respiratory*: PaO_2_/FiO_2_  ≤ 300 mmHg (40 kPa).

Organ failure is further characterised as transient, if evident for less than 48 hours or persistent, if longer. Thus, four categories of severity may be derived.

- *Mild acute pancreatitis* is characterized by the absence of both (peri)pancreatic necrosis and organ failure.
- *Moderate acute pancreatitis* is characterized by the presence of sterile (peri)pancreatic necrosis and/or transient organ failure.
- *Severe acute pancreatitis *is characterized by the presence of either infected (peri)pancreatic necrosis or persistent organ failure.
- *Critical acute pancreatitis* is characterized by the presence of infected (peri)pancreatic necrosis and persistent organ failure.

The classification of AP severity will continue to evolve and further modifications will be required in the future, driven by clinical experience and evaluation of the proposed new system.

Organ failure develops often early in the course of AP. About half of the patients who will develop organ failure will have it at admission or within 24 hours after admission [10–12]. The most common organ failure in severe AP is respiratory failure. In the presence of a single organ failure, mortality is less than 10%, whereas in multiorgan failure the mortality rate is 35–50% [1]. Organ failure may occur in the renal, hepatic, cardiovascular, digestive, neurologic, coagulation, endocrine, or immunologic system [13]. Also, failure of different organs has differing effects on prognosis [14]. If organ failure is already present on admission, this progresses to multiorgan failure in most of the patients and the mortality rate is high [11]. Indeed, half of the mortality takes place during the first week of the disease and is related to severe multiorgan failure [15]. The second peak of mortality occurs much later and is related to organ failure due to infectious complications and sepsis [15]. The duration of organ failure is also critical. If organ failure is determined as transient (<48 h), the patient will have a favourable outcome. In a case with persistent (>48 h) organ failure, the risk of morbidity and mortality is increased [16, 17]. Nevertheless, early identification of patients who develop a severe AP with organ failure would be essential to improve prognosis by earlier intervention with appropriate resuscitation in specialized hospitals.

At present, no specific medical treatment of AP exists. Treatment of the disease is mainly supportive and targeted to prevent and treat systemic complications. It is evident that delayed admission to intensive care unit worsens prognosis in patients with severe AP and early organ failure [18]. Indeed, prognosis of severe AP has improved due to early and aggressive conservative treatment in intensive care units. Early endoscopic retrograde cholangiopancreatography (ERCP) is recommended in the management of biliary AP with biliary obstruction [19]. Enteral feeding is considered to be a preferred method of delivering nutrition in severe AP and results in reduction of infectious complications and the need for surgery and lowers mortality rate [20, 21]. Later in the course of AP, infection complications are the major cause of morbidity and mortality. Therefore, prophylactic antibiotics have been used. However, serious concerns exist about a policy of antibiotic prophylaxis [22, 23]. In a study by Beger et al. carried out before antibiotic prophylaxis became widely used, organisms cultured from infected pancreatic necrosis were predominantly of gastrointestinal origin (*Escherichia coli* and *Bacteroides* spp.) [24]. The microbiology results of a later study, comparing perfloxacin and imipenem in pancreatic necrosis, were dominated by methicillin resistant *Staphylococcus aureus* and *Candida* spp. [25]. This development is important because evidence exists that indicates that infection with fungi and drug resistant organisms is associated with a significantly increased mortality [26]. Furthermore, results from two further randomised controlled trials fail to show a benefit for prophylaxis with antibiotics [27, 28]. The largest and most recent study of antibiotic prophylaxis was a multicenter, prospective, double-blind, and placebo-controlled randomized study set in 32 centres within North America and Europe [28] enrolled 100 patients with clinically severe, confirmed necrotizing pancreatitis: 50 received meropenem and 50 received placebo. This study demonstrated no statistically significant difference between the treatment groups for pancreatic or peripancreatic infection, mortality, or requirement for surgical intervention and did not support early prophylactic antimicrobial use in patients with severe acute necrotizing pancreatitis.

The role of probiotic therapy was evaluated by the Dutch Acute Pancreatitis Study Group [29]. The PROPATRIA trial was a double-blind, placebo-controlled, and randomised multicentre trial that aimed to show a reduction in infectious complications by the enteral use of a multispecies probiotic preparation in patients with predicted severe AP. The rationale for this study was clearly established. Infection of pancreatic necrosis is the major cause of death in (AP). Bacterial translocation across the gastrointestinal mucosal barrier is the mechanism thought to be responsible for this complication. Antibiotic prophylaxis has failed to reduce infectious complications. In contrast, probiotics are nonpathogenic bacteria that, on delivery to the host's intestinal tract, may reduce bacterial translocation by preventing bacterial overgrowth of pathogens, maintaining the gastrointestinal mucosal barrier, and by exerting local and systemic immunomodulatory effects. Thus, this was an eminently suitable topic for a randomised controlled trial. The finding of 15 excess deaths in this study in the probiotic group was unexpected and, indeed, shocking. Nine patients in the probiotics group developed bowel ischaemia (eight with fatal outcome), compared with none in the placebo group. However, the scientific evaluation of this new treatment may have saved many more lives; as it has prevented the *ad hoc* and widespread adoption of probiotic therapy, based on anecdote and personal bias, which would otherwise almost inevitably have occurred. This is precisely why randomised controlled clinical trials are performed. The mechanism of bowel ischaemia in the probiotics group remains a matter of further investigation. The administration of probiotic bacteria on top of enteral nutrition might have increased local oxygen demand, with a combined deleterious effect on an already critically reduced blood flow. A second possible explanation could be that the presence of probiotics caused local inflammation at the mucosal level. However, in view of the fatal nature of these complications, the administration of any type of probiotic in this category of patients must strongly be advised against.

Diagnosis of pancreatic necrosis is based on findings in dynamic contrast-enhanced computed tomography [30]. While determination of pancreatic necrosis requires contrast enhanced computed tomography, it may be inadvisable in a clinical emergency setting due to renal insufficiency and hypovolemia. Nowadays, it is recognized that, in terms of morbidity and mortality, organ failure is the most important factor [31, 32], regardless of the presence or absence of pancreatic necrosis, which develops later [33] and therefore the timing of contrast enhanced tomography may be delayed. The full extent of pancreatic necrosis cannot be appreciated until at least 3 days after symptom onset. It is recommended that patients with persisting organ failure, signs of sepsis, or clinical deterioration occurring after an initial improvement undergo CT scanning, which should be performed according to a pancreas protocol and all patients should receive oral and intravenous contrast [34].

Differentiation between sterile and infected necrosis is essential for those with >30% necrosis on CT and persistent symptoms or those with any degree of necrosis and signs of sepsis. This is achieved by fine needle aspiration for bacteriology of pancreatic or peripancreatic necrosis or the presence of retroperitoneal gas on CT [34, 35]. Patients with sterile necrosis should usually continue to be managed conservatively. The diagnosis of infected necrosis is an indication for radiological or surgical intervention.

Although good outcomes have been reported in patients with infected pancreatic necrosis managed by radiologically placed percutaneous drains, standard treatment remains surgical necrosectomy [34]. Novel minimal access approaches to necrosectomy have been described with particularly encouraging results obtained by a retroperitoneoscopic approach, combined with postoperative continuous irrigation [36]. A recent randomised controlled trial provided support for a “step-up” approach rather than primary open necrosectomy. This approach attempts to control sepsis with a radiologically placed drain and only if this is unsuccessful does the patient proceed to a necrosectomy. A minimally invasive approach is tried first, progressing to an open approach if sepsis does not fully resolve [37].

Generally, it is agreed that surgery should be postponed for as long as possible in AP. There exist, however, cases when intra-abdominal hypertension necessitates surgical decompression in an early phase of the disease [38].

## 3. Local and Systemic Inflammation

Irrespective of the etiological factor, triggering events of AP leads to a premature activation of pancreatic proteases as a result of intracellular colocalization of the digestive and lysosomal enzymes [2, 3]. Intracellular activation of pancreatic proenzymes leads to destruction of the parenchyma and autodigestion of the pancreas [2, 39–41]. It has recently been shown that autophagy (the principal cellular degradative pathway) in AP is activated but also impaired due to the defective function of lysosomes [42, 43]. Consequently, acinar cells become more prone to the deleterious effects of activated zymogens which will eventually lead to necrosis and inflammation [43, 44]. Also, there is an emerging body of evidence which suggests that the ubiquitous inducible transcription factor NF-*κ*B plays an important role in various stages of AP by mediating the expression of numerous genes involved in inflammation [4]. Since AP also affects extrapancreatic tissues, it was not surprising that NF-*κ*B activation could also be found outside of the pancreas. Although a link between pancreatic NF-*κ*B and trypsinogen activation in AP has been a matter of debate, recent results suggest that these processes may be unrelated and both can induce inflammation [4]. The earlier events may be mediated by intracellular Ca^2+^ signaling and reactive oxygen species [45].

Proinflammatory mediators in AP include various cytokines (e.g., tumour necrosis factor *α* (TNF-*α*), interleukin (IL)-1*β*, IL-2, IL-6, and IL-18), chemokines (e.g., IL-8, monocyte chemoattractant protein-1, macrophage inflammatory protein-1, monocyte chemoattractant protein-1, and growth-related oncogene-*α*), adhesion molecules, platelet-activating factor, and reactive-oxygen and reactive-nitrogen species [45–49]. In mild AP, local inflammation is controlled by the host's inflammatory response with localization of proinflammatory mediators in the affected area. In other severe cases, injury and inflammation in the pancreas can proceed to systemic inflammation causing systemic inflammatory response syndrome (SIRS) (Table 1) [50]. In some cases, this response is overwhelming and disseminated, when proinflammatory mediators, such as TNF-*α*, IL-1*β*, IL-6, and IL-8, are released into the circulation [51, 52]. In the liver, IL-6 is a potent inducer of synthesis of acute phase proteins, that is, C-reactive protein and procalcitonin [53]. Also, circulating neutrophils and monocytes become activated and express adhesion molecules (i.e., CD11b) and release their proteolytic enzymes and oxygen radicals, which damage vascular endothelial cells and organ parenchymal cells. Vascular endothelium is activated in the whole body and the expression of cellular adhesion molecules is upregulated which results in neutrophil extravasation and activation [54]. Endothelial permeability is enhanced leading to large amounts of tissue fluid (edema). This together with microvascular disturbances (i.e., vasoconstriction, inadequate perfusion, and increased blood viscosity) leads to lack of oxygen, which results in dysfunction and injury of vital organs [55, 56]. It has been demonstrated in experimental severe AP that microcirculatory disorders are present not only in the pancreas but also in the colon, liver, kidneys, and lungs [56]. In fact, lungs and kidneys are commonly injured organs in AP as they have an extensive capillary bed. Markers of hypovolemia (hemoconcentration, tachycardia, oliguria, and hypotension) are often seen in severe AP on admission.

The coagulation system is an integral part of the inflammatory response. It has been shown that coagulative disorders occur in severe AP [57–59] and they are related to severity of the disease and development of organ failure [60]. Systemic coagulation activation results in thrombosis in small and middle-sized vessels in many organs, which is called disseminated intravascular coagulation (DIC). Thrombocytopenia is a common sign of severe AP and is caused by excessive consumption of platelets in DIC. D-dimer is also a marker of DIC and has been shown to be high in severe AP [61]. Protein C is a natural anticoagulant in blood and has an essential role in the regulation of the coagulation cascade in inflammation. Protein C is activated by thrombin-thrombomodulin complex at the endothelial surface [62]. Activated protein C (APC) inactivates factor *V* and factor VIII and inhibits thrombin generation. APC also has anti-inflammatory effects in experimental studies [63, 64].

## 4. Compensatory Anti-Inflammatory Response Syndrome (CARS) and Immunosuppression

**Figure 1:**
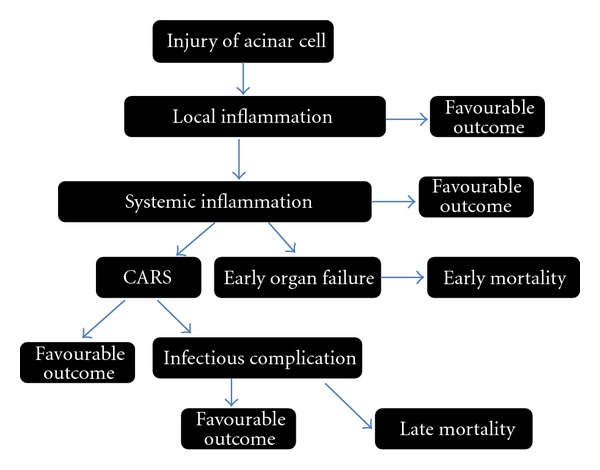
Inflammatory response in acute pancreatitis.

With the release of proinflammatory mediators, anti-inflammatory cytokines are concomitantly produced leading to a compensatory response syndrome (CARS) [65, 66]. High circulating concentrations of the anti-inflammatory mediators such as TNF-*α* receptors, IL-10, IL-11, and IL-1ra have been documented in AP [67–71]. If the anti-inflammatory response is adequate, the patient recovers. In a case of insufficient control, a proinflammatory burst leads to distant organ dysfunction. Anti-inflammatory reaction may also overcompensate and inhibit the immune response excessively rendering the patient susceptible to immunosuppression and infectious complications. Even though CARS happens in the early phase of severe AP, infectious complications are a result, at least partly, of impaired cellular immunity and occur in a later stage of the disease [24] (Figure 1).

Monitoring of HLA-DR expression is a useful marker for identifying monocyte function and is closely correlated with the clinical course in AP. In immunosuppression, defective host defence mechanisms include functional disturbances in monocytes which are characterized by a markedly reduced human leukocyte antigen (HLA)DR expression, and a diminished synthesis of proinflammatory cytokines, for example, TNF-*α* [72, 73]. IL-10, the most important anti-inflammatory cytokine, downregulates a number of proinflammatory cytokines [74]. In addition, it is able to decrease monocyte HLA-DR expression [75]. Monocytes with low HLA-DR density show impaired antigen presentation capacity [76]. IL-1ra is a specific antagonist to IL-1*β*, binds competitively to the IL-1 receptor, and blocks IL-1 mediated responses [77].

As early as 1989, Garcia-Sabrido et al. showed a correlation between poor outcome and anergy to delayed-type hypersensitivity testing as a marker of altered cellular immune function in AP [78]. Although IL-10 is an anti-inflammatory mediator, plasma IL-10 concentration is high in the very early phase of severe AP and is even a promising predictive marker of organ failure [70, 71]. Monocyte HLA-DR expression decreases at the early stage of severe AP [12, 79, 80]. Decreased monocyte HLA-DR expression predicts development of organ failure [12], development of secondary infections [66], and fatal outcome [80] in AP. There is a significant negative correlation of high plasma concentrations of IL-6 and IL-10 with HLA-DR expression in AP [66]. At present, the level of immunosuppression can be measured by laboratory means but this is not yet widespread in clinical practice. For example, chemiluminescent immunoassays are available for IL-10 and IL-6 but for monocyte HLA-DR measurement, flow cytometry is needed.

Li et al. recently investigated the expression of sphingosine kinase 1 (SphK1)/sphingosine 1-phosphate (S1P) in immune-effector cells, including neutrophils, monocytes, and lymphocytes, of 22 patients with severe AP in an effort to identify the role for SphK1/S1P in modulating the inflammatory response [81]. The expression of SphK1 and SphK activity was markedly increased in peripheral immune cells in the early stage of SAP and then reduced in the restoration stage in the patients. Moreover, they found that the level of S1P_3_ mRNA in peripheral neutrophils and lymphocytes of SAP patients was significantly elevated in the early stage as compared with the healthy volunteers, and it reduced in the restoration period. SphK1 expression on human peripheral neutrophils, monocytes, and CD4^+^ T lymphocytes were positively correlated with the APACHE II and levels of serum proinflammatory cytokines including TNF-(*α*), IL-1 (*β*), and IL-6. These observations show a possible immunomodulating role for SphK1/S1P signaling in inflammatory response in SAP, suggesting that regulation of SphK1/S1P pathway may represent novel targets in the treatment of SAP [81].

## 5. Immune-Modulation Therapy

At present, there is no specific medical therapy for AP. Patients with mild AP recover without intervention and novel treatment strategies should focus on patients with severe AP and a risk of organ failure. There is increasing evidence that in the early phase of AP, excessive leukocyte activation and inflammatory cytokine burst are critical for development of early organ failure and increased risk of MODS [82–85]. New therapeutic strategies attempting to prevent the activity of proinflammatory mediators or to block their synthesis have been evaluated as therapeutic options.

Progress in AP research is hampered by the inaccessibility of the human pancreas to observation, the lack of safe pancreatic biopsy techniques, difficulty in distinguishing initiating events from the concomitant inflammatory response, and the self-destructive nature of the disease process itself. Consequently, most of our knowledge about AP is derived from animal models of the disease, but these suffer from a lack of translational impact when applied to the human condition [86, 87]. Much remaining information results from studies of circulating inflammatory mediators and cells. Unsurprisingly, progress in the understanding and treatment of AP has been slow. The key to future advances must lie in obtaining data upon humans who have developed this disease, in comparison with meaningful controls.

One of the main interests has been TNF-*α*, which is the key regulator of other proinflammatory cytokines and a priming activator of immune cells [88]. In clinical studies, accurate evaluation of the role of TNF-*α* is problematic. Reasons explaining this general lack of correlation of this key inflammatory mediator with disease severity have concentrated on its short half-life, phasic release, the masking effects of circulating inhibitors, and its mainly paracrine level of function. It is important to appreciate that mainly tissue levels, not serum concentrations, are responsible for the vast majority of the biological effects of cytokines [4].

When given prophylactically or soon after the induction of experimental AP, anti-TNF antibodies decreased the severity of the disease in a rat [89] and mouse [90] model of AP, but there are also discouraging results [91]. Also, the clinical trials with anti-TNF in sepsis have not been successful [92, 93]. Blockage of the cytokine cascade at the level of the IL-1 receptor with its naturally occurring specific antagonist (IL-1ra) decreases pancreatic damage in caerulein-induced AP in mice [94]. Further, anti-inflammatory therapy with IL-10 agonist decreases the severity in a caerulein-induced AP in mice [95] and diminishes acute lung injury in a rabbit model of AP [96]. Selective inhibition of IL-1*β* in taurocholate-induced AP in rats [97] and inhibition of IL-8 in a rabbit model of AP [98] have shown beneficial effects. In experimental studies with mice, treatment with antibodies against adhesion molecules like intercellular adhesion molecule (ICAM)-1 has been effective [99–101]. Also, in a rat model of severe AP, endothelin receptor blockage was reported to reduce capillary leakage and improve microcirculation [102, 103]. In human studies, the use of a platelet-activating factor antagonist initially seemed promising in AP [104, 105], but later trials could not confirm the beneficial effects [10]. However, treatment attempts at blocking various single proinflammatory responses seem to be a flawed strategy. In the complex network of inflammatory response, a combination therapy to inhibit several proinflammatory agents may be more useful [106, 107]. Clinical trials of anti-inflammatory therapy has been difficult to conduct, as many of the patients present at a late stage of the disease, when organ failure is about to develop or may already be present [10]. It seems that the therapeutic window for anti-inflammatory therapies is very limited in clinical practice (for more details, see Section 6) [10] as the patient may be already on his way to CARS or even in immunosuppression.

Systemic inflammation and organ failure in severe AP show the same characteristics as the mechanisms induced by sepsis, major surgery, trauma, or severe burn [108]. Thus, research results from these conditions should be relevant to severe AP. For example, in sepsis, decreased protein C level in blood correlates with poor prognosis [109]. In severe AP, protein C pathway defects have been shown to be associated with development of organ failure [60]. In meningococcemia patients with DIC, treatment with APC has prevented development of organ failure and decreased mortality [110]. In patients with severe sepsis, treatment with APC was safe and was thought to result in decreased mortality [111, 112]. However, a recent randomized, double-blind, placebo-controlled, and multicenter trial has demonstrated that APC failed to significantly reduce mortality at 28 or 90 days in patients with septic shock [113]. In fact, due to the latter results, recombinant human APC was withdrawn from the market to treat sepsis by Eli Lilly in 2011. In a rat model of severe AP, APC treatment reduced inflammation in the pancreas and lungs and improved survival [114]. Recombinant human APC was also found to ameliorate cerulein-induced (mild) AP through apoptotic and NF-*κ*B pathways [115]. However, in a placebo-controlled clinical testing in 32 patients with severe AP, the APC treatment of severe AP did not bring clinical benefit for the patients [116].

Immunosuppression plays an important role in the development of secondary infections in the later course of AP. Treatment of patients with these late complications is a challenge with high mortality rates. Therefore, novel methods to diagnose and monitor the level of immunosuppression would be helpful. In clinical work therapeutic means to restore impaired host defence mechanisms would be helpful in patients with high risk of complications. Immunostimulation with interferon-gamma has proven to be beneficial in anergic septic patients [73]. In severe AP and sepsis, monocyte function is defective [73, 117]. Granulocyte-macrophage colony stimulating factor (GM-CSF) treatment was reported to increase monocyte HLA-DR expression and TNF-*α* production capacity and may also improve the clinical course in septic patients [118, 119]. *In vitro* study of monocytes taken from patients with AP showed that priming of cells by interferon-gamma and GM-CSF increases HLA-DR expression and restores lipopolysaccharide-induced TNF-*α* secretion [117]. These immunomodulatory therapies and the means to find the patients to benefit from them would be of utmost importance. However, further research must be done before optimal and individualized immunomodulatory treatment is possible.

 The interventional window: the optimal timing for delivering damage-limiting interventions was described by Norman [120]. This “interventional window” exists between the time of patient presentation and the onset of the development of organ dysfunction. Typically, the former occurs at 12–18 hours after disease onset whilst, for the latter, the incidence rises rapidly on the second and third day, distinguishing those likely to have a complicated attack from those likely to have a mild attack. Cytokine production begins shortly after disease onset but does not peak until 36–48 hours later. This has been elegantly demonstrated using post-ERCP pancreatitis as a human model to examine the initial cytokine response after the initiation of the disease [121]. This scenario provides a potential therapeutic window of opportunity that begins at hospital presentation and may last for 2-3 days, during which inflammatory mediator antagonism could be employed, in an attempt to attenuate the development of MODS. However, the experience of clinical trials, such as the phase III lexipafant (PAF antagonist) trial, challenge this [10]. In this investigation, the primary hypothesis, upon which the power calculation was based, was invalidated by the unexpected finding that 44% of patients had organ failure on entry into the study; only 14% developed new organ failure. 

## 6. Future Directions

Immunomodulation may represent a potential way to improve outcome in severe AP. However, it requires a thorough knowledge of underlying mechanisms and the patient's immunological state. At the moment we lack the essential information in order to modulate immune response, and more basic research is needed. Monitoring the state of immune dysfunction by monocyte HLA-DR expression during hospitalization of severe AP patients seems promising. Deeper understanding of the pathophysiology of AP is important to permit the design of effective interventions concerning the inflammatory response process. It is necessary to accurately identify patients with severe AP who are at risk of organ failure in order to transfer them urgently to an intensive care unit. Whether monitoring signaling pathways of circulating leukocytes, such as NF-*κ*B, signal transducers and activators of transcription (STATs), and members of mitogen activated protein kinase family helps us to find the patients at risk for secondary infections and, thus, late organ failure is at present under research [122–124]. Since multiple mediators are involved in the pathogenesis of AP, treatment strategies will probably focus on combination therapy in the future. Intuitively, it would seem helpful to depress the proinflammatory reaction in the patients at risk of excessive immune suppression so that inappropriate CARS would be prevented. However, it is evident that the window for anti-inflammatory therapy to suppress excessive activation of the inflammatory response is very limited. Finally, the analysis of signaling patterns of leukocytes may reveal novel therapeutic targets in severe AP.

## Figures and Tables

**Table 1:** Definitions of SIRS, sepsis, and MODS. Modified from American College of Chest Physicians/Society of Critical Care Medicine Consensus Conference. Definitions for sepsis and organ failure and guidelines for the use of innovative therapies in sepsis, 1992.

*Systemic Inflammatory Response Syndrome (SIRS)*: this response is manifested by two or more of the following conditions:	
Temperature >38°C or <36°C	
Heart rate >90 beats/min	
Respiratory rate >20 breaths/min or PaCO_2 _<4.3 kPa.	
White blood cell count <4,000 or >12,000 cells/mm^3^, or >10% immature (band) forms.	
*Sepsis*: this response is manifested when two or more of the above conditions occur as a result of infection.	
